# The Churches and Venereal Marriages: Do They Treat Marriage as a Sacrament or Not?

**Published:** 1917-12-08

**Authors:** G. Arbour Stephens


### THE EDITOR'S LETTER-BOX.

### The Churches and Venereal Marriages.

### Do They Treat Marriage as a Sacrament or Not?

*To the Editor of* THE HOSPITAL.

SIR,—I have read your article on "The Prophylaxis of Venereal Diseases" with much pleasure, and it seems to me to stand or fall on whether marriage is a sacrament or not.

As bearing on the subject the following case may be of interest: During my periodical examinations as factory surgeon I came across a young man suffering from syphilitic ulceration of the throat, quite unknown to himself save for a little hoarseness. I pointed out to him the serious condition of his bodily health, and also spoke to his father, who was employed in the same works. As a result of my talk he was under treatment for a year, when his father told me he was going to marry a decent young woman who was a Papist—the young man being a Protestant. Needless to say, I told his father that such a marriage was quite wrong under the circumstances, but I failed to make an impression—the only answer I could get being to the effect that they had bought the furniture.

The next step I took was to 'phone to the head priest of the woman's church and tell him the tale. His reply was to emphasise his impotency and declare *his inability to prevent the marriage.* If marriage is a sacrament, then a priest can prevent it; but if he cannot prevent it, then the only conclusion is that marriage is not a sacrament. Now, before this priest would perform this sacrament he would make it a condition that the *children* should be brought up Papists, so that, knowing the condition of this man, he was arranging for the propagation of syphilitic Roman Catholic children.

If this is the attitude of a big Church, what must be the attitude of the man in the street?—Yours,

G. ARBOUR STEPHENS, M.D.

